# Remarks upon the Report to Parliament of the National Vaccine Establishment, with Reasons in Favour of Vaccinating with One Puncture

**Published:** 1814-08

**Authors:** Edward Rigby

**Affiliations:** Norwich


*For the Medical and Physical Journal.*

### *Remarks upon the Report to Parliament of the National Vaccine Establishment, with Reasons in favour of Vaccinating with one Puncture; by* Mr. Edward Rigby.

IN the Report to Parliament of the National Vaccine Establishment, dated May 25, 1814, I am sorry to observe a paragraph which cannot fail to excite doubt and alarm in great numbers who have been vaccinated. It states that "most of the failures appear to have arisen from the practice of vaccinating with a single puncture, and afterwards opening the vesicle, and taking a portion of the lymph, for the purpose of propagating the infection."

No physiological reason is assigned for this, and I believe it would be difficult to prove that a single perfect vesicle, which goes through the usual stages and exhibits the characteric appearances of this singular disease, can be less the effect of a constitutional affection, than any given greater number would be; nor can it, I should think, be easily admitted that puncturing the vesicle, and abstracting a portion of the lymph, can have any tendency to destroy the constitutional principle which produced it. The ample experience I have had in vaccination has not in the smallest degree led me to suspect the possibility of such a circumstance. In Norwich and its neighbourhood it has been, hitherto, the general practice to vaccinate with one puncture only, and to take ichor in many instances: many thousands, I know, have been thus vaccinated; and, though in no place have the vaccinated been more exposed to variolous infection, in no place has vaccination been more completely successful. In my late Report of the Norwich Pauper Vaccination, from August 12, 1812, to August 12, 1813, I consider this fact to be fully established: I have there said, "though, during the vaccination from February to August, the small-pox still made a fatal progress, the melancholy fact afforded an irrefragible proof of the protracting power of vaccination: during this period probably not fewer than four hundred individuals had the small-pox; there was likewise no intermission of the disease, it was constantly spreading, and on many occasions, as before observed, patients were publicly exposed. Of the two thousand three hundred and ninety-one vaccinated during the year, it may be assumed, that at least two thousand have been resident in the city since February, and consequently equally exposed to an infectious atmosphere as the unvaccinated, and yet but one single instance, in that number, has occurred, in which the protecting influence of vaccination has been suspected, and this has been clearly ascertained to have been a case of premature vesicle, which suddenly rose, soon disappeared, and evidently produced no constitutional affection."*

It is ten months since this Report was written, and during this time the city has not been once free from the small-pox, and it still prevails in many of the neighbouring villages, and yet we hear of no failures in vaccination. I would also observe, that since vaccination was first introduced into Norwich, including a period of fifteen years, the small-pox has repeatedly appeared. It was very prevalent and very fatal in 1805 and 1806, and was again introduced in August 1807, and continued its ravages till the end of 1809; at this time the deaths from small-pox being recorded, it was ascertained that two hundred and three died, whence it is probable that more than twelve hundred individuals had the disease. In 1812 it was again admitted, by an infected

* See Med. Journ. vol. xxxi. p. 138.

child child coming from the country, and subsequently by variolous inoculation; but the prompt and extensive vaccination which at that time took place arrested its progress, and the city was again free from it till February 1813, as before noticed.*

It is obvious that a great majority, not only of the paupers who have been more recently vaccinated, but of those of all classes who have been vaccinated in Norwich within the last fifteen years, must necessarily have been exposed to the contagion of small-pox, at one or other of these periods, and yet, I repeat it, we have heard of no failures in vaccination.

From this ample testimony it cannot surely be doubted that a single perfect vesicle affords as complete security against variola as any indefinite number; and, if so, there would seem to be an obvious objection to unnecessarily multiplying the vesicles, which in all cases go through a high degree of inflammation, are often attended with painful tumefaction and even suppuration in the axilla, and, if exposed in the latter stage to any act of violence, are apt to assume a very disagreeable ulceration; more especially as young children, now the principal subjects of vaccination, are most liable to suffer in this way.

In making two punctures, it must be acknowledged there is a double chance of the lymph being absorbed; and there certainly are situations in which it may be right to profit of this, as in country practice, where the patient is at a considerable distance from the surgeon, and failure in the first instance is obviously attended with much inconvenience, and the facility of obtaining fresh ichor being there so much less than in towns, where vaccination is more constantly going on.

Having thought it right to vaccinate all my patients with one puncture, and having taken ichor from many of them, I feel it incumbent on me, in this public manner, most unequivocally to declare my perfect conviction of its security.

*Norwich, July* 4, 1814. EDWARD RIGBY.

---

*For the Medical and Physical Journal.*

### *Observations on an indigenous Remedy for Cancer, with a Copy of the Prescription; by* LEONARD GILLESPIE, M.D.

THAT savage nations are possessed of secrets, as to the nature of plants and their virtues, powerful in destroying and in preserving life, the testimony of many enlightened

* See Facts relating to the Poor in Norwich, p. 92 and 93.

